# Phytochemistry, nutritional composition, health benefits and future prospects of *Mespilus germanica* L. (Medlar): A review

**DOI:** 10.1016/j.fochx.2024.101334

**Published:** 2024-03-31

**Authors:** Doru Ion Nistor, Romina Alina Marc, Crina Carmen Mureșan

**Affiliations:** Food Engineering Department, Faculty of Food Science and Technology, University of Agricultural Sciences and Veterinary Medicine, 400372 Cluj-Napoca, Romania

**Keywords:** Antibacterial, *Mespilus*, *M. germanica*, Medlar, *Rosaceae*

## Abstract

*Mespilus germanica* L., commonly known as medlar, is one of two species of the Rosaceae family. The medlar plant has a long history of use in gastronomy and healthcare. Medlar waste is used to extract hazardous heavy metals from contaminated water. The nutritional value of *M. germanica* fruits comes from their composition of carbohydrates, carotenoids, amino acids, organic acids, proteins, vitamins, fatty acids, and vital components. *M. germanica* fruit contains a high concentration of important phenolic components, which contribute to its anti-diabetic and antioxidant properties. Additionally, several studies have identified diverse biological properties of the *M. germanica* plant, including the cytotoxic, neurodegenerative, and antibacterial properties of its fruits and leaves. Scientists are investigating underutilized plant species to address sustainability issues in food production. This review study will provide a comprehensive examination of its chemical composition, medical applications, plant waste utilization, and potential biological activities.

## Highlights

• Traditional uses, Commercial uses and Medicinal uses of *M. germanica*.
• Uses of wastes produced by Medlar.
• Antioxidant, antidiabetics, cytotoxic, neutodegenerative activities by M germanica (Medlar).
• Chemical composition of *M. germanica* L.
• Compound classes (organic acids, sugars, Volatile compounds, Fatty acids, vitamins, phenols) found in *M. germanica*.

## Graphical abstract

**Figure:**
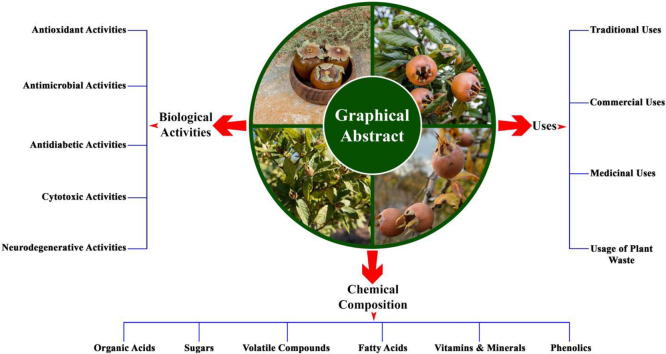

## 1 Introduction

Rosaceae is a large family of flowering plants belonging to the Rosales order, comprising about 100 genera, 16 tribes, and around 3000 species worldwide (Voronkov, Kumachova, & Ivanova, 2020; Xiang et al., 2016; Zhang et al., 2017). These plants are mainly found in Northern Hemisphere temperate forests, where they play a crucial role in providing habitat and food for various animals (Xiang et al., 2016). The family consists of three subfamilies, namely Rosoideae, Dryadoideae, and Amygdaloideae. The Amygdaloideae subfamily is a combination of the previous Maloideae, Amygdaloideae, and Spiraeaoideae subfamilies (Kumachova et al., 2023; Voronkov, Kumachova, & Ivanova, 2020; Xiang et al., 2016). *Mespilus* L. is a genus of the Pyrinae subtribe, which belongs to the Amygdaloideae subfamily (Kumachova et al., 2023; Potter et al., 2007). This genus has two known species, namely *Mespilus canescens* J. B. Phipps and *Mespilus germanica* L., commonly called the common medlar (Voaides et al., 2021). *M. germanica* was first discovered in North America in 1990, while *M. canescens* is native to Southeastern Europe and Southwest Asia.(Akbulut et al., 2016; Bibalani & Mosazadeh-Sayadmahaleh, 2012).

**Fig. 1:**
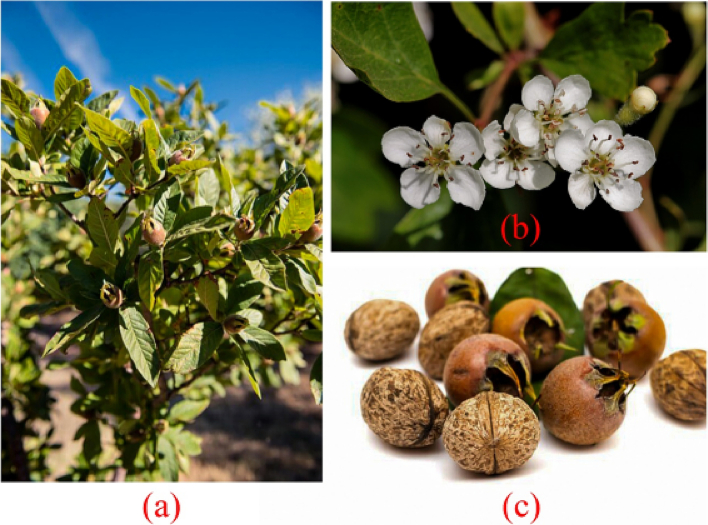
*Mespilus germanica*. (a): Plant overview. (b): Flower. (c): Fruit.

The *M. germanica* is a deciduous shrub with thorns (sometimes without thorns) and has simple, elliptical or oblong-lanceolate leaves (Fig. 1) (Kumachova et al., 2023). The young leaves are glabrous with trichomes on both sides, deep green on the top and light green on the bottom, with greater pubescence around the central vein (Haciseferoǧullari et al., 2005; Kumachova et al., 2023). The edges of the fully formed leaf are pointed at the tip, notched, and have reddish-brown glandular appendages (Kumachova et al., 2023; Żołnierczyk et al., 2021). The fruits of *M. germanica* are harvested during autumn, after the first frost (Cosmulescu et al., 2020; Cosmulescu & Scrieciu, 2020). Its fruit contains fatty acids and has high antioxidant capacity (Canbay et al., 2011; Cosmulescu & Scrieciu, 2020; Nabavi et al., 2011). *M. germanica* fruits are healthy but lose their taste after a few weeks. Freshly harvested or overripe fruits can turn brown and gooey. However, overripe fruits have a delicious and mildly acidic texture that can be consumed at this stage (Voaides et al., 2021). *M. germanica* fruits are sensitive to climate, and white fruits cannot be eaten due to high tannin levels (Assefa et al., 2020; Koçyiǧit et al., 2016; Shafiee et al., 2018; Żołnierczyk et al., 2021). To soften the fruit, some of the October harvest is stored in a dark, cool, and well-ventilated environment. Pickled *M. germanica* fruits are a popular winter snack (Glew, Ayaz, Sanz, et al., 2003a; Glew, Ayaz, Sanz, et al., 2003b; Glew, Ayaz, Vanderjagt, et al., 2003); Voaides et al., 2021). (See Table 1, Table 2.)

**Table 1:** The medical uses of *M. germanica*.

Disease Name	Plant part used	Region of usage	References
Abdominal pain	Bark	Turkiye	(Koçyiǧit et al., 2016)
Anti-Lithiasis	Leaves, Fruits	Algeria, Turkiye	(Bouabdelli et al., 2012; Koçyiǧit et al., 2016; Popovic-Djordjevic et al., 2023)
Antithelmintic	Bark	Turkiye	(Koçyiǧit et al., 2016)
Asthema	Fruit, Leaves	Turkiye	(Güneş et al., 2017)
Cholera	Fruits	Iran	(Bibalani & Mosazadeh-Sayadmahaleh, 2012)
Cold, Flu	Fruit, Leaves	Turkiye, Iran	(Bibalani & Mosazadeh-Sayadmahaleh, 2012; Kültür, 2007)
Cough	Leaves	Turkiye	(Kültür, 2007; Miser-Salihoglu et al., 2010)
Cutaneous Ieishmaniasis	Leaves	Iran	(Bibalani & Mosazadeh-Sayadmahaleh, 2012)
Diabetes	Leaves	Turkiye	(Koçyiǧit et al., 2016; Miser-Salihoglu et al., 2010)
Diarrhea	Fruit, Leaves, Bark	Turkiye, Iran, Serbia	(Miser-Salihoglu et al., 2010; Kültür, 2007; Bibalani & Mosazadeh-Sayadmahaleh, 2012; Jarić et al., 2015; Paksoy, Selvi, & Savran, 2016)
Diuretic	Fruits	Iran	(Bibalani & Mosazadeh-Sayadmahaleh, 2012)
Dryness	Fruits, Leaves	Iran	(Bibalani & Mosazadeh-Sayadmahaleh, 2012)
Dynestery	Fruit, Leaves	Iran	(Ahvazi & Akbarzadeh, 2017)
Enteritis	Pulp	Turkiye	(Koçyiǧit et al., 2016)
Eyesight issues	Fruits	Serbia	(Jarić et al., 2015)
Fever	Wood	Iran	(Cevahir & Bostan, 2021)
Hematopoietic	Fruit, Leaves, Bark	Iran	(Popovic-Djordjevic et al., 2023)
Hemorrhoids/Internal Hemorrhage	Leaves	Iran, Turkiye	(Bibalani & Mosazadeh-Sayadmahaleh, 2012; Miser-Salihoglu et al., 2010)
Hepatitis	Fruit, Leaves	Turkiye	(Güneş et al., 2017)
Hypoglycemia	Leaves, Fruits	Algeria, Iran	(Ahvazi & Akbarzadeh, 2017; Bouabdelli et al., 2012)
Inflammations	Fruit	Turkiye	(Miser-Salihoglu et al., 2010)
Influenza	Fruit, Leaves	Turkiye	(Sargin, 2021)
Large intestine infection	Fruit, Leaves, Bark	Iran	(Popovic-Djordjevic et al., 2023)
Liver and kidney issues	Fruits	Serbia	(Jarić et al., 2015)
Menstrual irregularities	Fruits	Iran	(Popovic-Djordjevic et al., 2023)
Nerve strengthening	Fruits	Iran	(Bibalani & Mosazadeh-Sayadmahaleh, 2012)
Obesity	Fruits	Iran	(Bibalani & Mosazadeh-Sayadmahaleh, 2012; Popovic-Djordjevic et al., 2023)
Oral abscess	Leaves	Iran	(Bibalani & Mosazadeh-Sayadmahaleh, 2012)
Rheumatism	Leaves	Turkiye	(Miser-Salihoglu et al., 2010)
Skin strengthening	Leaves	Iran	(Bibalani & Mosazadeh-Sayadmahaleh, 2012)
Stomach bloating, Stomachache, Constipation	Fruits, Leaves	Turkiye, Iran	(Koçyiǧit et al., 2016; Ahvazi & Akbarzadeh, 2017; Bibalani & Mosazadeh-Sayadmahaleh, 2012)
Throat infections	Leaves	Iran	(Bibalani & Mosazadeh-Sayadmahaleh, 2012)
Vomiting	Fruit, Leaves	Iran	(Ahvazi & Akbarzadeh, 2017)

**Table 2:** Overview of the heavy metals adsorbed by the Medlar waste products.

Heavy Metal	Limit of metal tolerance	Effect of heavy metal	Medlar waste type	Technique used	Maximum adsorption capacity	Reference
Chromium (Cr)	0.005 ppm	Renal diseases, Kidney damage, Cancer-causing	Seeds	AC from seed for metal adsorption	200 mg/g	(Solgi et al., 2017)
Cadmium (Cd)	0.01 ppm	Carcinogenic, Renal problems, Plant growth and grain loss.	Medlar peels	Biosorption	98.14 mg/g	(Benaïssa, 2006)
Copper (Cu^2+^)	0.01 ppm	Affects blood clotting. Melena, arterial hypertension, Wilson diseases, digestive discomfort, sleeplessness.	Medlar fruit core	Carbonized medlar-core particles used as biosorbent	43.478 mg/g	(Langeroodi & Safaei, 2016)
Nickel (Ni^2+^)	2 μg/L	Cause pulmonary embolism, liver damage, asthma, chronic bronchitis.	Medlar leaves	AC from leaves used for metal adsorption	13.08 mg/g	(Khedri et al., 2022)

*M. germanica* also known as Medlar, is a versatile plant that has a range of ornamental, medicinal, and dietary uses (Cosmulescu & Scrieciu, 2020; Popovic-Djordjevic et al., 2023). Its fruits can be eaten raw or cooked and can be used to make a variety of products such as cheese, jam, juice, honey and leather goods (Popovic-Djordjevic et al., 2023; Salehi, 2020). Premature fruits can be used to make pickles or vinegar. The fruit is a good source of nutrients such as carotenoids, vitamins, organic acids, sugars, fatty acids, amino acids, and vital components. Medlar fruits, leaves, wood, and bark have been used in traditional medicine, and the fruit flesh is known to have stimulant properties. (Bibalani & Mosazadeh-Sayadmahaleh, 2012; Davoodi et al., 2018; Shafiee et al., 2018).

Numerous scientific articles have extensively studied *M. germanica* from various angles, such as pomological, phylogenetic, phenotypic, chemical composition, antioxidant activities, antibacterial characteristics, ripening stage influence, and medicinal benefits (Bouabdelli et al., 2012; Niu et al., 2013; Ahmady-Asbchin et al., 2013; Tabatabaei-Yazdi et al., 2015; Selcuk and Erkan, 2015a, Selcuk and Erkan, 2015b; Canbay et al., 2011; Tabatabaei-Yazdi et al., 2015; Akbulut et al., 2016; Davoodi et al., 2017; Safari & Ahmady-Asbchin, 2019; Ozturk et al., 2019; Isbilir et al., 2019; Cevahir & Bostan, 2021; Tessa et al., 2021; Denizkara et al., 2021; Stankovic et al., 2022). *M. germanica* has been recognized for about three millennia, from the ancient Babylonians and Assyrians to the Romans and Greeks (Akbulut et al., 2016). It is said that this fruit, which is not native to Europe, was imported during the Roman era. Over time, the fruit gained popularity, as evidenced by textual materials and artworks that prominently featured the medlar (Popovic-Djordjevic et al., 2023). Despite its previous fame, *M. germanica* is now frequently ignored or neglected fruit across Europe (Akbulut et al., 2016).

Medlar is a fruiting plant that grows naturally in various regions, including the Transcaucasus and Caucasus mountains, Asia Minor, northern Iran, southern Crimea, Greece and the Balkan peninsula (Bibalani & Mosazadeh-Sayadmahaleh, 2012; Davoudzadeh et al., 2018). Azerbaijan has the most diverse varieties, with some wild variants found in Turkmenistan (Popovic-Djordjevic et al., 2023; Voaides et al., 2021). *M. germanica* has been cultivated for thousands of years in temperate areas of Anatolia (Atay, 2013). This plant was likely cultivated some 3000 years ago in Northern Iran's Caspian Sea region and modern-day Turkey's Black Sea beaches (Haciseferoǧullari et al., 2005). It was brought to Greece around 700 BCE and to Rome around 200 BCE. It appears to have been a popular fruit plant throughout the medieval and Roman periods (Voaides et al., 2021). However, it fell out of favor during the 17th and 18th centuries, but it is now being cultivated again (Voaides et al., 2021). There was no information on manufacturing this plant anywhere in the world, not even in the countries where it is produced (Voaides et al., 2021). Nowadays, Medlar is hardly grown and can only be found in home lawns and botanical gardens (Sebek et al., 2019; Solomonova et al., 2019; Voaides et al., 2021). However, it is widely cultivated in Turkey, Germany, and the Netherlands (Bostan, 2002; Haciseferoǧullari et al., 2005; Sebek et al., 2019; Voaides et al., 2021; Yilmaz et al., 2016).

In recent years, scientists have been focusing on neglected and underutilized plant species that are used in decorative and gardening environments. These plants could help solve the problems of sustainability in food and agricultural production. With the world population increasing, global warming, and natural resources being depleted, these plant varieties could play a crucial role in boosting natural resources, helping the agricultural industry withstand environmental difficulties, and providing a basis for healthy meals. In addition, these plants can be used to enhance decoration and landscaping efforts. One such promising fruit plant is *M. germanica*, which offers many nutritional and physiological benefits. The purpose of this review study is to provide a comprehensive examination of the chemical composition, traditional uses, medical uses, commercial uses, plant waste use, and potential biological activities of *M. germanica*. The study only considers works that have made significant contributions to this field of research in the last twenty years.

## 2 Uses of *M. germanica*

### 2.1 Traditional uses

**Fig. 2:**
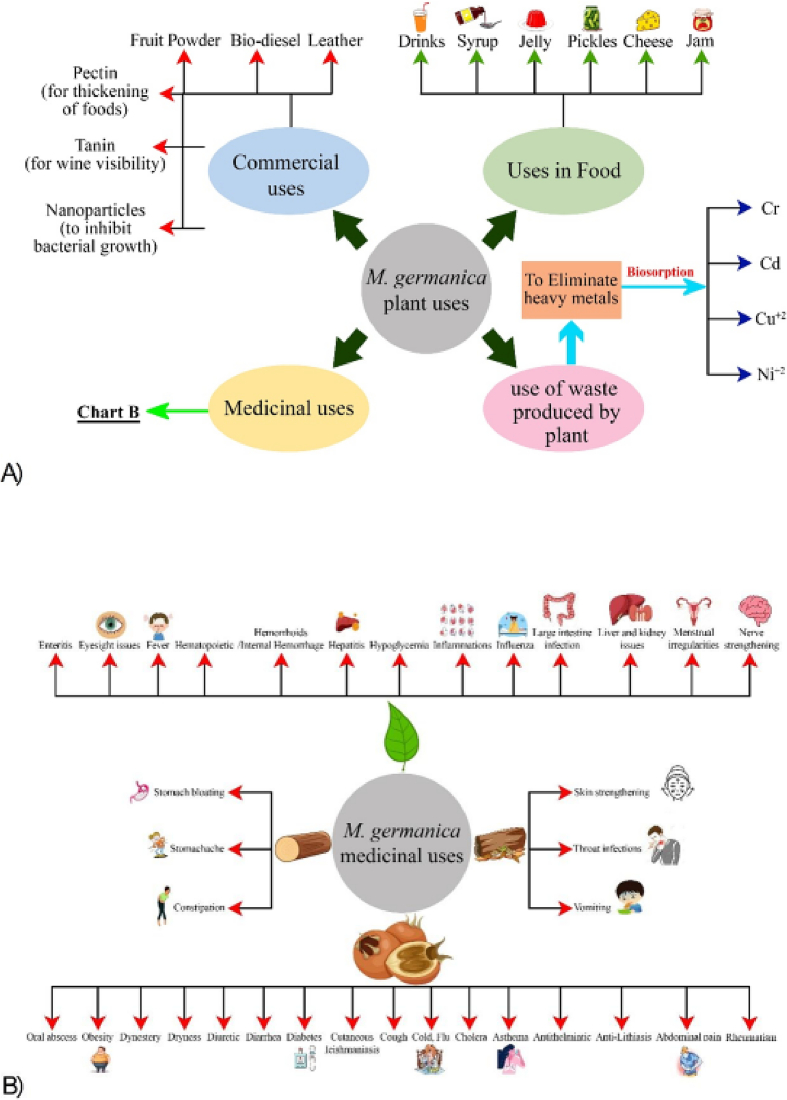
(A): Overall uses of *M. germanica*. (B): The potential medicinal uses of *M. germanica*.

*M. germanica* (medlar), has a wide range of traditional applications. It can be consumed in various forms, such as fresh, vinegar, pickled, boiled, crushed, or dried-out pulp (Bostan, 2002). It is also used as a remedy for various ailments, mainly constipation and renal and urinary tract issues (Suna, 2019; Yue et al., 2021). However, its most common application is raw consumption (Ozturk et al., 2019; Salehi, 2020). The mature fruits of *M. germanica* can be eaten as is or processed into a diverse range of products, including juice, beverages, sauces, jelly, cheese, jams, leather, and syrup. (Fig. 2A) (Bibalani & Mosazadeh-Sayadmahaleh, 2012; Ozturk et al., 2019; Rop et al., 2011; Salehi, 2020; Suna, 2019; Yue et al., 2021). Unripe fruits can also be used to make pickles and beverages such as cider (Bibalani & Mosazadeh-Sayadmahaleh, 2012; Popovic-Djordjevic et al., 2023). Additionally, the fruits are used to make a specific jelly that can be used as a pie filling. Medlar cheese, which is a type of lemon curd, is also made using fruit paste, margarine, and eggs (Popovic-Djordjevic et al., 2023).

### 2.2 Commercial uses

Medlar fruit, a climacteric fruit, has gained importance as a popular food item among humans, which has increased its economic value. As a result, it is now available in large stores in metropolitan centres and regional outdoor markets (Fig. 2A). Researchers are interested in revealing its biological and nutritive features due to its commercial significance (Glew, Ayaz, Sanz, et al., 2003a; Glew, Ayaz, Sanz, et al., 2003b; Glew, Ayaz, Vanderjagt, et al., 2003). Medlar fruit powder can be used to enhance certain biophysical and physiological aspects of sponge biscuits fortified with this powder (Uçar & Hayta, 2018). The inclusion of powder increases the stiffness and lifespan of the cookies and also raises the crumb's redness value. Moreover, medlar fruit powder boosts the overall phenolic profile of the cake while improving its antioxidant qualities (Uçar & Hayta, 2018). Medlar is also an excellent source of pectin, which is a natural polysaccharide used as a functional ingredient in the food industry for thickening, increasing viscosity, forming gels, and modifying flavors (Al-Amoudi et al., 2019; Tabatabaei-Yazdi et al., 2015). The pectin extracted from medlar fruit has a high level of methoxyl pectin and primarily includes ᴅ-glucose, L-rhamnose, ᴅ-galacturonic acid, ᴅ-galactose, and L-arabinose (Al-Amoudi et al., 2019; Aydin & Kadioglu, 2001; Cevahir & Bostan, 2021).

Medlar fruit has a reasonably significant tannin concentration of around 3% and produces flocculation of proteins, making it useful for lowering wine visibility (Davoodi et al., 2018). *M. germanica* kernel oil was used to create bio-diesel using an Al_2_O_3_/CaO nano-catalyst. The extracted oil's primary ingredients were 41% oleic acid and 43% linoleic acid (Foroohimanjili et al., 2020), making it a potential substitute for diesel fuels without requiring any modifications to traditional machinery (Qasemi et al., 2022). Additionally, silver nanoparticles produced from *M. germanica* extract showed anti-biofilm and antibacterial properties against *Klebsiella pneumoniae* clinical isolates with resistance to multiple drugs (Foroohimanjili et al., 2020).

### 2.3 Medicinal uses

Medicinal plants are known to contain significant amounts of secondary metabolic products, including essential oils, antifungal agents, antibiotic substances, and other molecules. These properties make them potential substitutes for synthetic drugs (Bibalani & Mosazadeh-Sayadmahaleh, 2012; Canbay et al., 2011; Cevahir & Bostan, 2021; Donno et al., 2017; Ercisli et al., 2012; Javaid et al., 2022; Nabavi et al., 2011; Popovic-Djordjevic et al., 2023; Tessa et al., 2021). Studies have shown that herbal medications are effective, have no adverse effects, and are inexpensive compared to synthetic medications. As many as 80% of people worldwide rely on traditional medical practices for their basic healthcare (Bibalani & Mosazadeh-Sayadmahaleh, 2012; Süntar, 2020). The production of indigenous medicines and the therapeutic use of medicinal species for the treatment of multiple illnesses has significant financial benefits (Astutik, Pretzsch, & Kimengsi, 2019; Aziz et al., 2018; Javaid et al., 2023; Süntar, 2020). However, with the advancements in science, synthetic medications have taken over, leading to the decline of herbal remedies (Astutik et al., 2019; Gilani & Atta-ur-Rahman., 2005). Nonetheless, herbal medications are still frequently recommended due to their efficacy and low cost (Anand et al., 2019; Hameed et al., 2011; Süntar, 2020).

The *M. germanica* plant is widely used for medicinal purposes in several countries, including Turkey, Iran, Algeria, and Serbia (Bibalani & Mosazadeh-Sayadmahaleh, 2012; Bouabdelli et al., 2012; Davoodi et al., 2017; Kültür, 2007; Tabatabaei-Yazdi et al., 2015). Different parts of the plant are used for therapeutic purposes, such as the leaves, bark, fruits, and wood. *M. germanica* is known to cure a variety of ailments, such as diarrhea, oral abscess, stomach bloating, throat abscess, obesity, fever, hematopoietic, internal hemorrhage, cholera, constipation, throat ailments, skin issues, nerves issues, intestinal inflammation, large intestine infection, irregular menstrual cycle, and cutaneous leishmaniasis (Fig. 2B) (Al-Ishaq et al., 2019; Bibalani & Mosazadeh-Sayadmahaleh, 2012; Donno et al., 2017; Msomi et al., 2021; Popovic-Djordjevic et al., 2023; Tessa et al., 2021; Yildiz et al., 2023; Żołnierczyk et al., 2023). The fruit of the plant is also believed to have health benefits such as improving vision, kidney, and liver health, and treating renal and urinary tract stones (Jarić et al., 2015; Koçyiǧit et al., 2016; Popovic-Djordjevic et al., 2023; Tabatabaei-Yazdi et al., 2015).

Infusions of medlar fruit are used to treat various conditions, including colitis, diarrhea, internal bleeding, cholera, menstrual irregularities, obesity, abdominal spasms, colds, and dehydration. The infusion is also known to have haematological, nerve-stimulating, and diuretic qualities (Bibalani & Mosazadeh-Sayadmahaleh, 2012). Nectar derived from *M. germanica* fruit is used to treat enteritis (Gülçin et al., 2011; Nabavi et al., 2011; Popovic-Djordjevic et al., 2023; Tessa et al., 2021). Decoctions of medlar leaf, along with the fruit, are used to treat coughing, influenza, colds, diarrhea, joint inflammation, diabetes, bleeding disorders, infections of the large intestine, internal hemorrhage, skin leishmaniasis, hypoglycemia, and oral infection (Bibalani & Mosazadeh-Sayadmahaleh, 2012; Davoodi et al., 2018; Popovic-Djordjevic et al., 2023; Żołnierczyk et al., 2023). *M. germanica* leaf is also used for haematological, skin-tightening, throat therapy, and anti-lithiasis purposes (Bouabdelli et al., 2012; Cevahir & Bostan, 2021; Popovic-Djordjevic et al., 2023). Additionally, infusions made from the leaves and fruits of *M. germanica* are widely used to alleviate various health issues such as stomach pain, diarrhea, nausea, hypoglycemia, respiratory difficulties, hepatitis, and pneumonia (Ahvazi & Akbarzadeh, 2017; Güneş et al., 2017; Jarić et al., 2015; Sargin, 2021; Żołnierczyk et al., 2021). The powdered form of medlar wood and bark is beneficial in treating major intestinal infections, diarrhea, internal hemorrhage, and fever, and also has haematological and diuretic properties (Bibalani & Mosazadeh-Sayadmahaleh, 2012; Popovic-Djordjevic et al., 2023; Sadeghinejad et al., 2022). It is believed that a decoction made from medlar bark can help alleviate stomach discomfort as it possesses anthelmintic properties (Davoodi et al., 2018; Pető et al., 2016; Popovic-Djordjevic et al., 2023). Although there is limited research on the therapeutic benefits of *M. germanica*, different parts of the plant are believed to serve different therapeutic purposes.

### 2.4 Uses of wastes produced by medlar

Fruit waste is generated during the cleaning and sorting processes in fruit production activities (Bhardwaj et al., 2022). There are two types of waste produced during fruit growth: solid waste (seeds, peels, stones, etc.) and liquid waste (juices and their wash fluids) (Sha et al., 2023). Fruit waste is a rich source of secondary metabolites, which can be used to make food additives, preservatives, nutritional supplements, activated carbon sources for heavy metal ions removal, and biological adsorbents for wetland restoration (Benaïssa, 2006; Bhardwaj et al., 2022;Khedri et al., 2022; Langeroodi & Safaei, 2016; Solgi et al., 2017). Waste products from fruits and vegetables are rich in bioactive chemicals and are considered the most basic form of functional nutrients (Sha et al., 2023). Fruit waste that is high in flavonoids and polyphenols has antioxidant properties and can reduce the risk of developing several malignancies (Day et al., 2009). The literature has identified several important applications of Medlar plant waste (Fig. 2A). Medlar seeds can be used to produce activated carbon, which can remove heavy metals from contaminated water (Benaïssa, 2006). The peels, fruit cores, and leaves of medlar plants can be used as biosorbents to remove harmful metal ions from wastewater and aquatic streams (Khedri et al., 2022; Langeroodi & Safaei, 2016; Solgi et al., 2017).

Activated carbon is a highly versatile material used in various processes, such as air filtration, power generation, and wastewater treatment. It is especially useful in removing hazardous heavy metals from water (Abioye & Ani, 2015). Activated carbon is produced through carbonization, which can be chemically or physically activated. This process involves using different carbonaceous substances, such as farm and natural wastes, vegetable and fruit waste products, and other types of waste (Abioye & Ani, 2015; Kumar et al., 2018; Shehzad et al., 2015; Skodras et al., 2007). Solid fruit wastes like medlar seeds, palm shells, orange peels, grape seeds, bananas, and pomegranate peels are frequently used in producing activated carbon for water pollution removal (Elias et al., 2021; Issabayeva et al., 2006; Ukanwa et al., 2020). Another organic waste that can generate activated carbon is *M. germanica* seed waste, which has a significant potential for producing activated carbon. Solgi et al. (2017) studied a novel activated carbon produced from *M. germanica* seeds, for the removal of chromium Cr (VI). This was chemically stimulated with KOH and carbonized at temperatures ranging from 450 to 750 °C. The highest adsorption capacity for Cr (VI) on activated carbon derived from *M. germanica* seeds was 200 mg/g (Solgi et al., 2017).

Adsorption is an efficient, cost-effective, and eco-friendly method for purifying water (Sharma, Vyas, & Dalai, 2017). Agricultural and food waste can be used as biosorbents to remove toxic metal ions from wastewater, and this innovative approach is gaining popularity. Using waste materials for heavy metal removal is not only cost-effective but also highly effective (Ayranci & Duman, 2009; Bhardwaj et al., 2022; Khedri et al., 2022; Langeroodi & Safaei, 2016; Paredes et al., 2015; Periyasamy et al., 2020; Solgi et al., 2017). Research has been conducted to remove heavy metals such as arsenic, cadmium, lead, mercury, chromium, nickel, cobalt, and others using different types of biosorbents (Benaïssa, 2006; Khedri et al., 2022; Langeroodi & Safaei, 2016; Periyasamy et al., 2020; Pohl et al., 2013). Medlar peel waste was found to be more effective in cadmium removal at an acidic pH (Benaïssa, 2006; Periyasamy et al., 2020). The efficiency of adsorption is affected by the contact period, the initial cadmium concentration, and the type of sorbent used. Medlar peel showed a maximum sorption capacity of 98.14 mg/g, successfully reducing cadmium ions (Benaïssa, 2006).

*M. germanica* core in carbonized form is a highly efficient biosorbent for removing Copper (Cu^2+)^ from aqueous solutions (Langeroodi & Safaei, 2016). The percentage of adsorption of Cu^2+^ is influenced by operational factors such as adsorbent dosage, solution pH, temperature, and initial adsorbent concentration. The maximum adsorption capacity of carbonized medlar core for Cu^2+^ is 43.478 mg/g, which is better than other natural sorbents, including unprocessed pinecone (38.46 mg/g), activated sludge (65.789 mg/g), and sawdust (1.79 mg/g) (Adeyemo et al., 2014; Langeroodi & Safaei, 2016; Wu et al., 2012; Yu et al., 2000). Moreover, the leaves of *M. germanica* can be used to create activated carbon for removing Nickel (II) (Ni2+) from water-based solutions (Khedri et al., 2022). The adsorbent's surface area is 10.39 m^2^/g, and its maximum adsorption rate is 13.08 mg/g. The optimum nickel ion adsorption rate is 97.56% at pH 7, temperature 298 K, and a starting concentration of 60 ppm nickel ions (Khedri et al., 2022).

## 3 Potential biological activities of medlar plant

### 3.1 Antioxidant activities

Numerous investigations have been carried out to evaluate the antioxidant properties of different parts of *M. germanica* (Table 3). Various in vitro experiments, such as DMPD^+^ (*N,N*-dimethyl-*p*-phenylenediamine), Superoxide anion radicals scavenging activity, Ferrous ion chelating activity, ABTS (2,2- azino-bis-3-ethylbenzothiazoline-6-sulfonic acid), DPPH (2,2-diphenyl-1-picrylhydrazyl), Cupric ion reducing antioxidant capacity (CUPRAC), Ferrous ion reducing ability, total antioxidant capacity (TAC), β-carotene bleaching, and Ferric reducing antioxidant power (FRAP), have demonstrated that *M. germanica* fruit possesses significant antioxidant properties (Akbulut et al., 2016; Canbay et al., 2011; Foroohimanjili et al., 2020; Gülçin et al., 2011; Haciseferoǧullari et al., 2005; Miser-Salihoglu et al., 2010; Stankovic et al., 2022; Voaides et al., 2021; Voronkov, Kumachova, & Ivanova, 2020; Żołnierczyk et al., 2021; Żołnierczyk et al., 2023). The antioxidant activity of medlar genotypes varied (Akbulut et al., 2016; Canbay et al., 2011; Ercisli et al., 2012; Stankovic et al., 2022; Yildiz et al., 2023).

**Table 3:** Antioxidant activities reported for the *M. germanica*.

Biological activity	Plant part used	Assay	Extraction solvents	Reference
Antioxidant activity	Fruit	SOD enzyme activity	Phosphate buffer	(Campanella et al., 2003)
	Fruit, Leaves, Bark, Buds	DPPH radical scavenging activity	Methanol and distilled water. The chemical compounds present are acetone, water, formic acid, and methanol. The chemical compounds present are acetone, water, acetic acid, ethanol, and a mixture of hydrochloric acid, methanol, and water.	(Ercisli et al., 2012; Isbilir et al., 2019; Selcuk & Erkan, 2015a; Suna, 2019; Cevahir & Bostan, 2021; Nabavi et al., 2011; Ozturk et al., 2019; Stankovic et al., 2022; Gülçin et al., 2011; Gruz et al., 2011; Safari & Ahmady-Asbchin, 2019; Selcuk & Erkan, 2015b)
	Fruit	DMPD and O2 radicals scavenging activity	Distilled water	(Gülçin et al., 2011)
	Fruit, Leaves, Bark	NO and H2O2 radicals scavenging activity	Methanol or water	(Nabavi et al., 2011; Popovic-Djordjevic et al., 2023)
	Fruit	ABTS radical scavenging assay	HCl:Methanol:Water, Chloroform: methanol, Ethanol: water: HCl	(Stankovic et al., 2022; Rop et al., 2011; Żołnierczyk et al., 2021; Akbulut et al., 2016; Canbay et al., 2011)
	Fruit, Leaves, Bark	Fe chelating activity, Fe reducing ability	Distilled water, Methanol or water	(Gülçin et al., 2011; Nabavi et al., 2011; Popovic-Djordjevic et al., 2023)
	Fruit	FRAP	Distilled water, Water, Aqueous methanol, Ethanol: water: HCl, HCl:methanol: water	(Gülçin et al., 2011; Ozturk et al., 2019; Suna, 2019; Tessa et al., 2021; Żołnierczyk et al., 2021)
	Fruit	CUPRAC	Distilled water, HCl:methanol: water	(Gülçin et al., 2011; Suna, 2019)
	Fruit, Leaves, Buds	β-carotene bleaching	Acetone: water: acetic acid, Ethanol	(Ercisli et al., 2012; Isbilir et al., 2019)
	Fruit	TAC scavenging activity	Ethanol	(Stankovic et al., 2022)

Additionally, the choice of extraction solvent had a significant impact on the antioxidant properties of *M. germanica* fruit. Methanol is a superior solvent for extracting antioxidants when compared to water, according to certain studies. The study analyzed the water and methanol components of frozen *M. germanica* fruit and determined that the methanol extract exhibited the highest antioxidant potential (Nabavi et al., 2011; Qasemi et al., 2022; Żołnierczyk et al., 2021). When evaluating the antioxidant characteristics of *M. germanica* fruit, it is essential to take into account the stage of ripeness. The antioxidant capacity of *M. germanica* fruit is evaluated using the ABTS^+^ and DPPH assays, which demonstrate a noticeable decline during the maturity process (Gruz et al., 2011; Popovic-Djordjevic et al., 2023; Rop et al., 2011; Sebek et al., 2019). According to published research, the antioxidants in overripe *M. germanica* fruit were more than twice as abundant as those with ripe fruit harvested just 10 days later (Gruz et al., 2011).

Medlar fruit is rich in health-promoting antioxidants, which is why it is best to consume it as soon as it ripens. In experiments measuring antioxidant capacity, 1 g of medlar fruit extract was found to be as effective as 238.2 mg of ascorbic acid. Although it had lower antioxidant activity compared to blackthorn, medlar fruit extract had higher activity than hawthorn and blackthorn fruit extracts in vitro (Stankovic et al., 2022). In addition, medlar fruit has been found to have a higher antioxidant capacity than figs, plums, cherries, grapes, apples, apricots, pineapples, melons, pears, bananas, watermelons, and peaches (Campanella et al., 2003). Research has also been carried out to determine the impact of various storage conditions on the antioxidant capacity of medlar fruit. Fruits stored in an enhanced atmospheric packaging system had higher antioxidant activity than fruits stored in a controlled environment, as determined by the DPPH scavenging technique (Selcuk & Erkan, 2015a).

However, after 60 days of storage, the antioxidant activity decreased. Another study found that exposure to 1-methylcyclopropene helped medlar fruits maintain an elevated amount of antioxidant capacity, which subsequently decreased during storage (Selcuk & Erkan, 2015b). Altering the packaging environment, either individually or in conjunction with *Aloe vera* gel or methyl jasmonate, greatly slowed the depletion of antioxidant activity during the preservation of medlar fruits (Ozturk et al., 2019). Finally, the impact of different drying methods, including microwave, hot air, and vacuum drying, on the antioxidant properties of medlar fruit was investigated using DPPH, FRAP, and CUPRAC assays (Selcuk & Erkan, 2015a).

Various drying methods were used to dry *M. germanica* fruits and their impact on the fruit's antioxidant capacity was studied using in vitro digestion (Popovic-Djordjevic et al., 2023). Although all drying methods reduced the fruit's antioxidant capacity, microwave heating resulted in the highest antioxidant preservation. The study found that the antioxidant properties of dried *M. germanica* fruits improved after digestion as seen in the results of the FRAP and DPPH experiments. However, the findings of the CUPRAC test did not support these conclusions (Popovic-Djordjevic et al., 2023). It is widely recognized in the scientific literature that a single experiment cannot adequately quantify the antioxidant capacity. Therefore, it is recommended to use a variety of assays employing different methodologiesto obtain a more comprehensive understanding (Capanoglu et al., 2017; Isbilir et al., 2019; Jalali et al., 2022; Voaides et al., 2021). Many studies have investigated the antioxidant potential of *M. germanica* buds, leaves, bark, and fruit. The leaves of *M. germanica* have remarkable antioxidant qualities due to their capacity to effectively neutralize DPPH radicals (Popovic-Djordjevic et al., 2023; Safari & Ahmady-Asbchin, 2019; Voaides et al., 2021).

The potency of antioxidants of *M. germanica* fruit, bark, and leaf extracts was found to be the most potent of all evaluated materials (Nabavi et al., 2011), and the researchers observed that the radical-scavenging ability of each components increased with dosage. In addition, the antioxidant level of several sections of the *M. germanica*, including the fruit, leaves, and floral buds, was evaluated through DPPH and β-carotene bleaching tests (Isbilir et al., 2019), and the leaf extract was found to have the highest antioxidant activity in both trials compared to buds and fruits (Popovic-Djordjevic et al., 2023).

### 3.2 Antimicrobial activities

Limited studies have been conducted on the antimicrobial properties of the *M. germanica* plant, as shown in Table 4 (Bouabdelli et al., 2012; Denizkara et al., 2021; Foroohimanjili et al., 2020; Niu et al., 2013; Safari & Ahmady-Asbchin, 2019; Tabatabaei-Yazdi et al., 2015). In an investigation to determine its antibacterial activity, the *M. germanica* fruit extracts were tested against *Klebsiella pneumoniae* and *Staphylococcus aureus* (Niu et al., 2013). The scientists discovered that the fruit extracts of *M. germanica* were somewhat susceptible to *S. aureus* but had a strong inhibitory impact on *Klebsiella pneumoniae*. Moreover, the ethanol-prepared fruit extract of *M. germanica* outperformed the water extract in terms of antibacterial activity (Niu et al., 2013). Similarly, another study compared the development of *Listeria innocua*, *Streptococcus pyogene*, *Klebsiella pneumoniae*, and *Enterobacter aerogenes* using the fruit extract of *M. germanica* (Tabatabaei-Yazdi et al., 2015). The results showed that the extract had a stronger inhibitory effect on the development of *Listeria innocua* and *Streptococcus pyogene* compared to *Klebsiella pneumoniae* and *Enterobacter aerogenes*. This indicates that the extract has a bigger antibacterial impact on Gram-positive bacteria than Gram-negative bacteria. Additionally, when the fruit extract of *M. germanica* was combined with widely used pharmaceutical antibiotics, it prevented the growth of more strains of bacteria (Tabatabaei-Yazdi et al., 2015).

**Table 4:** Antimicrobial activities reported for *M. germanica*.

Plant part used	Class of Microorganism	Name of microorganism against which the Disc diffusion technique used	Solvent used	Reference
Fruits	Fungi	*Aspergillus flavus*, *A. niger*	Ethanol, methanol, acetone or water	(Denizkara et al., 2021)
Fruits	Bacteria	*Bacillus cereus*	Ethanol, methanol, acetone or water	(Denizkara et al., 2021)
Leaves	Bacteria	*Citrobacter freundii*	Methanol	(Safari & Ahmady-Asbchin, 2019)
Fruits	Bacteria	*Enterobacter aerogenes*	Water or ethanol	(Tabatabaei-Yazdi et al., 2015)
Leaves	Bacteria	*Enterococcus faecalis*	Methanol	(Safari & Ahmady-Asbchin, 2019)
Fruits, Leaves	Bacteria	*Escherichia coli*	Water or ethanol, Aqueous acetone, Methanol	(Ahmady-Asbchin et al., 2013; Bouabdelli et al., 2012;Davoodi et al., 2017; Denizkara et al., 2021; Safari & Ahmady-Asbchin, 2019)
Fruits	Bacteria	*Klebsiella pneumoniae*	Water or ethanol, Aqueous acetone, Methanol	(Niu et al., 2013)
Fruits	Bacteria	*Listeria innocua*, *L. monocytogenes*	Water or ethanol, Methanol, Acetone	(Denizkara et al., 2021; Tabatabaei-Yazdi et al., 2015)
Fruits	Fungi	*Mucor racemosus*	Ethanol, methanol, acetone or water	(Denizkara et al., 2021)
Fruits	Fungi	*Penicillium notatum,* *P. crysogenum*	Ethanol, methanol, acetone or water	(Denizkara et al., 2021)
Leaves	Bacteria	*Proteus mirabilis*	Water	(Bouabdelli et al., 2012)
Leaves	Bacteria	*Pseudomonas aeruginosa*	Water, Methanol, Ethanol	(Ahmady-Asbchin et al., 2013; Bouabdelli et al., 2012; Safari & Ahmady-Asbchin, 2019)
Fruits	Fungi	*Rhizopus nigricans*	Ethanol, methanol, acetone or water	(Denizkara et al., 2021)
Leaves	Bacteria	*Salmonella typhi*, *S. paratyphi*	Ethanol, methanol, acetone or water	(Safari & Ahmady-Asbchin, 2019)
Leaves	Bacteria	*Serratia marcescens*	Methanol	(Safari & Ahmady-Asbchin, 2019)
Fruits, Leaves	Bacteria	*Shigella dysenteriae*	Ethanol, methanol, acetone or water	(Davoodi et al., 2017; Denizkara et al., 2021; Safari & Ahmady-Asbchin, 2019)
Fruits	Bacteria	*Staphylococcus aureus*, *S. epidermidis*	Ethanol, methanol, acetone or water	(Niu et al., 2013)
Fruits	Bacteria	*Streptococcus pyogene*	Ethanol, methanol, acetone or water	(Tabatabaei-Yazdi et al., 2015)
Leaves	Bacteria	*Vibrio cholera*	Aqueous acetone	(Davoodi et al., 2017)
Leaves	Bacteria	*Yersinia enterocolitica*	Methanol	(Safari & Ahmady-Asbchin, 2019)

A recent study examined the antibacterial properties of *M. germanica* fruit extracts using various solvents such as acetone, methanol, water, and ethanol (Denizkara et al., 2021). The extracts were tested against several bacteria and fungi using the disc diffusion method. The results showed that the water extracts had the highest antibacterial efficacy against *Listeria monocytogenes*, *S. aureus*, *Shigella dysenteria*, *S. enterica* ser. *Typhimurium*, *Bacillus cereus*, *Escherichia coli*, *Aspergillus niger*, *Aspergillus flavus*, *Penicillium crysogenum*, *Penicillium notatum*, *Rhizopus nigricans*, and *Mucor racemosus*. The results indicated that water extracts had the highest level of antibacterial efficacy (Denizkara et al., 2021). In addition, four distinct water extractions from *M. germanica* leaves were analyzed for their antibacterial capabilities against *Proteus mirabilis*, *E. coli*, *Pseudomonas aeruginosa*, and *S. aureus*. The study found that *M. germanica* leaf infusion and decoction had high antibacterial action (Bouabdelli et al., 2012).

In various studies, researchers have examined the use of extracts from *M. germanica* leaves for their antibacterial properties. In one study, ethanolic and methanolic extracts were tested against *P. aeruginosa*, *E. coli*, and *S. aureus*, which are commonly found in healthcare environments (Ahmady-Asbchin et al., 2013). The results showed that methanolic extracts were more effective in suppressing the growth of these bacteria as compared to ethanolic extracts (Ahmady-Asbchin et al., 2013). In another study, water-acetone extracts from *M. germanica* leaves were tested against several bacteria, such as *Shigella dysenteriae*, *E. coli*, *Vibrio cholerae*, and *K. pneumoniae*. The results showed that the extract was particularly effective against *K. pneumoniae* (Davoodi et al., 2017). In yet another supplementary study, the antibacterial activity of different concentrations of methanol extract from medlar leaves was evaluated against various bacteria such as *Staphylococcus epidermidis, S. aureus, P. aeruginosa, Serratia marcescens, E. coli, Streptococcus pyogenes, K. pneumoniae, Salmonella typhi, Yersinia enterocolitica, Salmonella paratyphi, Enterococcus faecalis, Citrobacter freundii* and *Shigella dysenteriae*. The highest level of inhibitory activity was observed against *S. aureus* (Safari & Ahmady-Asbchin, 2019). Overall, the studies have demonstrated that leaf extracts of *M. germanica* have greater antibacterial action against Gram (+ive) bacteria than Gram (−ive) bacteria. Additionally, the antibacterial efficacy of this leaf extract was found to be greater than that of some bacteria, such as *E. coli*, *S. epidermidis*, and *S. aureus* (Safari & Ahmady-Asbchin, 2019).

### 3.3 Antidiabetic activities

Several studies have been carried out to investigate the effectiveness of various components of the *M. germanica* plant in treating diabetes. An in vitro experiment showed that an ethanolic extract of *M. germanica* fruit had better inhibition of α-glucosidase, compared to the conventional antidiabetic medication acarbose (Stankovic et al., 2022). The inhibitory effects of α-glucosidase and α-amylase enzymes have been observed in many components of the *M. germanica* plant, particularly the flower buds, leaves, and fruits (Isbilir et al., 2019). All extracts of *M. germanica* were found to inhibit α-glucosidase, while the bud and fruit extracts had inhibitory effects on pancreatic α-amylase of swine. Among the tested extracts, flower bud extracts showed the most inhibition for α-glucosidase and α-amylase. According to the scientists, the inhibition might be due to the phenolics found in *M. germanica* (Isbilir et al., 2019). Another study reported that the water and methanolic fraction of the *M. germanica* extract have anti-diabetic properties (Żołnierczyk et al., 2021).

It has been found that only a small portion of the water-based extract of *M. germanica* has significant anti-diabetic benefits (Żołnierczyk et al., 2023). The flavonoid content of its leaves has been reported to dramatically lower blood levels of insulin, TNF-a, and glucose in rats with ovarian cancer (Kouhestani et al., 2018; Yunusa & Ozturk Urek, 2023). Studies have also shown that *M. germanica* leaf extract is effective in reducing blood glucose levels, reducing oxidative stress, and stabilizing body weight in both healthy and diabetic rats (Shafiee et al., 2018). In fact, in diabetic rats, the plant leaf extract administered orally significantly reduced blood glucose levels, lipid peroxidation, and oxidative stress. Additionally, it helped maintain body weight, surpassing the effects of metformin. Another study also found that *M. germanica* can reduce blood glucose levels and decrease apoptotic markers in diabetic rats. In particular, the rats treated with *M. germanica* showed lower levels of caspase-8 and caspase-3 compared to the control group (Popovic-Djordjevic et al., 2023).

### 3.4 Cytotoxic activities

The study evaluated the cytotoxicity of *M. germanica* fruit extract on three human cancer cell lines: cervical adenocarcinoma, malignant cell line, and colon adenocarcinoma. (Yunusa & Ozturk Urek, 2023). The extract showed potent cytotoxic action against cervical cancer cells, with an IC50 rate of 624.83 μg/mL, which was the highest observed. The extract had minimal efficacy against malignant cells, with an IC50 value of 854.98 μg/mL. However, it did not demonstrate any cytotoxic effects against colon cancer cells (Stankovic et al., 2022). Furthermore, the study investigated the effects of *M. germanica* leaf extract on the growth and development of carp fingerling skin, as well as its impact on non-specific immune markers in the skin mucus and antioxidant genes (Hoseinifar et al., 2017). The addition of medlar leaf extract to fingerlings enhanced their growth rate, regardless of the dosage. Additionally, this extract treatment improved the activity of antioxidant enzymes that are controlled by genes in the epidermis (Hoseinifar et al., 2017).

### 3.5 Neurodegenerative activities

The study examined the effects of an extract from *M. germanica* leaves on male Wistar rats induced with streptozotocin, specifically in terms of cognitive impression, instruction, and memory retention (Darbandi et al., 2018). The results showed that the administration of Streptozotocin injection significantly impaired cognitive function, memory retention, and the integrity of CA1 neurons in comparison to the control group. However, when supplemented with medlar flavonoid extract, cognitive functions were significantly enhanced, and memory was preserved. This was demonstrated by an increase in the number of viable cells in the hippocampus CA1 area, accompanied by a decrease in the number of dead cells (Darbandi et al., 2018). Additionally, the flavonoid extract derived from the leaves of *M. germanica* was found to mitigate memory impairment induced by amyloid β-42 in rats, due to its role in reducing cytochrome C levels (Davoudzadeh et al., 2018).

## 4 Chemical composition of *M. germanica* L

Humans rely on various natural compounds, particularly fruits, that are rich in antioxidants and can help prevent various diseases (Donno et al., 2017; Żołnierczyk et al., 2023). Fruit consumption has traditionally been shown to improve human health, and dietary fibre is an excellent addition to dietary guidelines as they are a great source of antioxidants, minerals, and vitamins (Slavin & Lloyd, 2012; Yasin et al., 2020). Given the apparent importance of antioxidants in health, research has been expedited to discover new antioxidant alternatives and analyze existing antioxidant resources (Kaviani Livani et al., 2018; Llauradó Maury et al., 2020; Xu et al., 2017). Additionally, phenols and lipids are essential for the fruit's fragrance, flavour, and nutritional significance (Sadeghinejad et al., 2022). As a result, there has been a surge in demand for wild fruits in recent decades, primarily due to the growing awareness of their vitamin and mineral content, potent medicinal effects, and unique flavors (Khoo et al., 2016).

*M. germanica* is a plant that is rich in natural phytochemicals, but it has been largely ignored and understudied by the scientific community due to its low production. However, individuals across southeastern Europe, Turkey, Iraq, Siberia, and Iran have long recognized its value (Popovic-Djordjevic et al., 2023). The fruit of *M. germanica* contains a high concentration of organic acids, pectins, amino acids, carotenoids, sugars, trace elements, minerals, and polyphenols, making it nutritionally significant (Al-Amoudi et al., 2019; Ayaz et al., 2008; Popovic-Djordjevic et al., 2023; Sadeghinejad et al., 2022; Voaides et al., 2021; Żołnierczyk et al., 2021). The fruit also contains bioactive substances such as fatty acids and phenols fatty acids and phenols (Sadeghinejad et al., 2022; Stankovic et al., 2022; Yildiz et al., 2023), which make it a valuable source of organic antioxidants that can be used in culinary and medicinal products (Akbulut et al., 2016; Donno et al., 2017; Khadivi et al., 2019; Yildiz et al., 2023; Żołnierczyk et al., 2021).

There is significant variation in medlar species when it comes to fruit-related features in different locations, has allowed for the selection of trees with both high-quality fruits and productivity (Khadivi et al., 2019; Sadeghinejad et al., 2022; Voaides et al., 2021). However, information on its biological characteristics and chemical constituents is scarce. Nutritional analysis of *M. germanica* has focused on mineral content, carotenoids, vitamins, sugar content, fatty acids, proteins, macroelements and microelements (Azizi & Azizi, 2011; Donno et al., 2017; Ercisli et al., 2012; Pető et al., 2016; Sadeghinejad et al., 2022; Sebek et al., 2019; Żołnierczyk et al., 2021). Fluctuations in nutrient levels across *M. germanica* cultivars can be related to cultivar variances, environmental variables, developmental stage, and soil fertility (Ercisli et al., 2012; Pető et al., 2016; Popovic-Djordjevic et al., 2023; Sadeghinejad et al., 2022; Voaides et al., 2021; Żołnierczyk et al., 2021).

Recently, researchers have recently become interested in the bioactive chemicals found in *M. germanica*, specifically flavonoids and phenolic acids (Akbulut et al., 2016; Gruz et al., 2011; Gülçin et al., 2011; Ozturk et al., 2019; Popovic-Djordjevic et al., 2023; Rop et al., 2011; Stankovic et al., 2022; Żołnierczyk et al., 2021). In addition to phenolics, its fruit contains vitamins, organic acids (tartaric, citric, oxalic, malic etc.), fatty acids, sugars (pectin, sucrose, fructose, glucose etc.), minerals, volatile compounds, and proteins (Akbulut et al., 2016; Al-Amoudi et al., 2019; Cevahir & Bostan, 2021; Cosmulescu et al., 2020; Ercisli et al., 2012; Glew, Ayaz, Sanz, et al., 2003a; Glew, Ayaz, Sanz, et al., 2003b; Glew, Ayaz, Vanderjagt, et al., 2003; Haciseferoǧullari et al., 2005; Pető et al., 2016; Popovic-Djordjevic et al., 2023; Tessa et al., 2021; Veličković et al., 2013; Voaides et al., 2021; Voronkov, Ivanova, et al., 2020; Żołnierczyk et al., 2021). Table 5 outlines the research linked to the chemical configuration of *M. germanica* and the analytical methodologies used.

**Table 5:** Overall scenario of the chemical composition of the *M. germanica* reported in the literature.

Compound Class	The technique used for extraction	Chemical Compounds obtained	Reference
Organic acids	HPLC–DAD, HPLC-RI, HPLC–UV, GLC,	Tartaric acid, Ascorbic acid Oxalic acid, Fumaric acid, Citric acid, Malic acid, Succinic acid, Fumaric acid, Quinic acid, Malic acid	(Cevahir & Bostan, 2021; Cosmulescu et al., 2020; Cristofori et al., 2019; Ozturk et al., 2019; Selcuk & Erkan, 2015a; Tessa et al., 2021; Voaides et al., 2021)
Fatty acids	GC–MS, GC-FID	Oleic acid, Lingoceric acid, Arachidic acid, Behenic acid, Stearic acid, Palmitic acid, Palmitoleic acid, Myristic acid, Lauric acid, Cerotic acid, Linoleic acid, Margaric acid, Phthalic acid	(Azizi & Azizi, 2011; Glew, Ayaz, Sanz, et al., 2003a; Glew, Ayaz, Sanz, et al., 2003b; Glew, Ayaz, Vanderjagt, et al., 2003)
Amino acids	HPLC	Aspartate, Glutamate	(Glew, Ayaz, Sanz, et al., 2003b; Glew, Ayaz, Vanderjagt, et al., 2003; Popovic-Djordjevic et al., 2023)
Ascorbic acid	HPLC-RI, HPLC-UV, LC-MS/MS, HPLC-DAD, HPLC-DA, HPLC-ED, HPLC/UV	Ascorbic acid, Dehydroascorbic acid	(Cevahir & Bostan, 2021; Cosmulescu et al., 2020; Glew, Ayaz, Sanz, et al., 2003a; Glew, Ayaz, Sanz, et al., 2003b; Glew, Ayaz, Vanderjagt, et al., 2003; Gülçin et al., 2011; Ozturk et al., 2019; Rop et al., 2011; Selcuk & Erkan, 2015a; Tessa et al., 2021)
Phenolics	UHPLC/HESI, HPLC/DAD, MS/MS, UPLC/DAD, LC-MS/MS, HPLC/UV–Vis	Protocatechuic acid, Quercetin, Coumaric acid, Aesculetin, Rutin, Kaempferol, Chlorogenic acid, Pinocembrin, Syringic acid, Caffeic acid, Catechins, Gallic acid, Benzoic acids: Ellagic acid, Cinammic acid, Ferulic acid, Epicatechin, Neochlorogenic acid, Procyanidin B, Sinapic acid, Vanillin, Resveratrol, Pyrogallol	
			(Akbulut et al., 2016; Gruz et al., 2011; Gülçin et al., 2011; Ozturk et al., 2019; Rop et al., 2011; Sadeghinejad et al., 2022; Stankovic et al., 2022; Tessa et al., 2021; Żołnierczyk et al., 2021)
Sugars	GLC, HPLC-RI, HPLC/UV	Fructose, Glucose, Sucrose, Sorbitol	(Cevahir & Bostan, 2021; Cristofori et al., 2019; Glew, Ayaz, Sanz, et al., 2003a; Glew, Ayaz, Sanz, et al., 2003b; Selcuk & Erkan, 2015a)
Minerals	ICP-AES, Atomic Absorption Spectrometry	S, Ba, Na, Cd, Ni, Cr, Fe, In, K, Al, Li, Ca, Mg, B, Mn, Pb, Mo, P, Se, V, Cu, Zn, As, Co, Ti, Sr,	(Cevahir & Bostan, 2021; Ercisli, 2007; Ercisli et al., 2012; Glew, Ayaz, Vanderjagt, et al., 2003; Haciseferoǧullari et al., 2005; Pető et al., 2016)
Volatile compounds	GC–MS	Monoterpenes (Phellandrene, γ-terpinene, terpinolene), Esters, Terpenes, C6 aldehydes (hexanal, furfural, and (Z)-3-hexanol), and (*E*)-2-hexenal) and alcohols (hexanol), Benzaldehyde, Pentadecane, Tetradecane	(Pourmortazavi et al., 2005; Tessa et al., 2021; Veličković et al., 2013)
Other compounds	LC-MS/MS, HPLC/UV–Vis	α-tocopherol, β-carotene, Vitamin C	(Gülçin et al., 2011; Olives Barba et al., 2006; Popovic-Djordjevic et al., 2023)

### 4.1 Compound classes found in *M. germanica*

#### 4.1.1 Organic acids

**Fig. 3:**
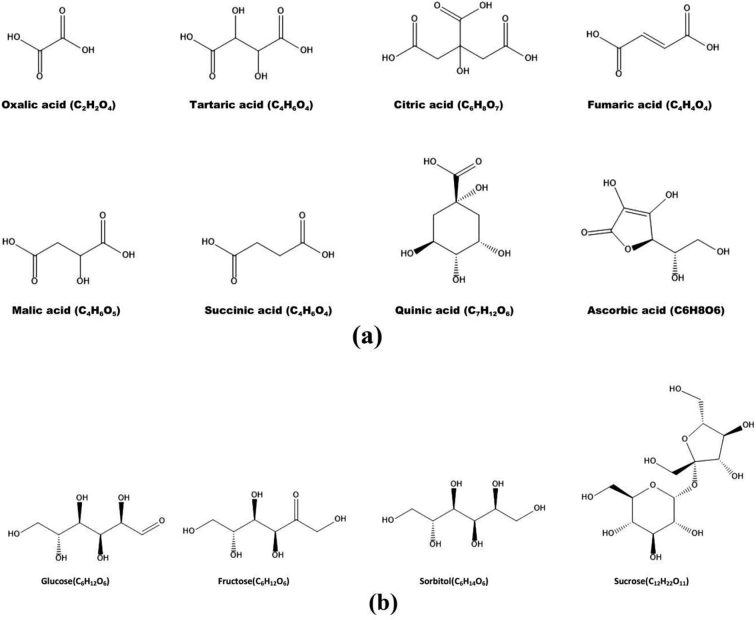

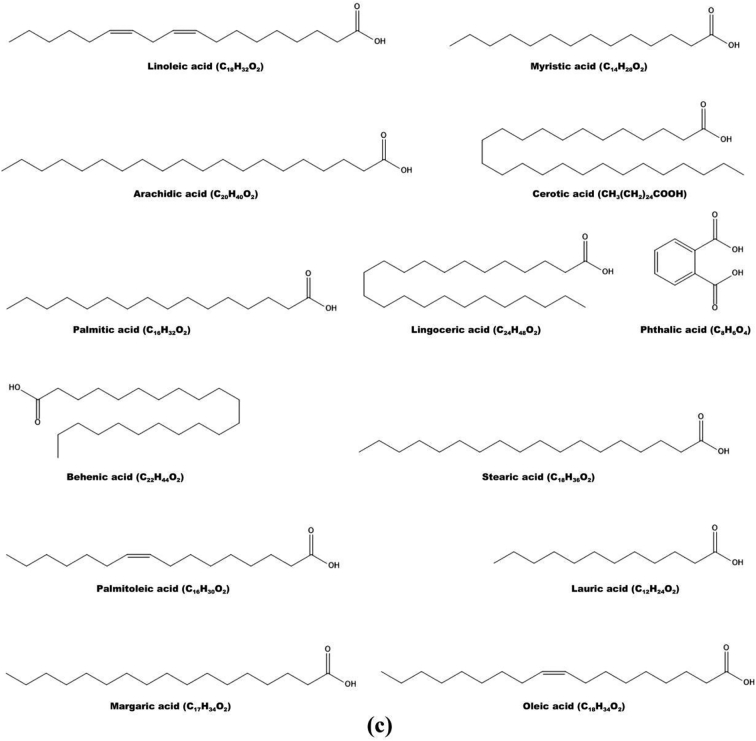

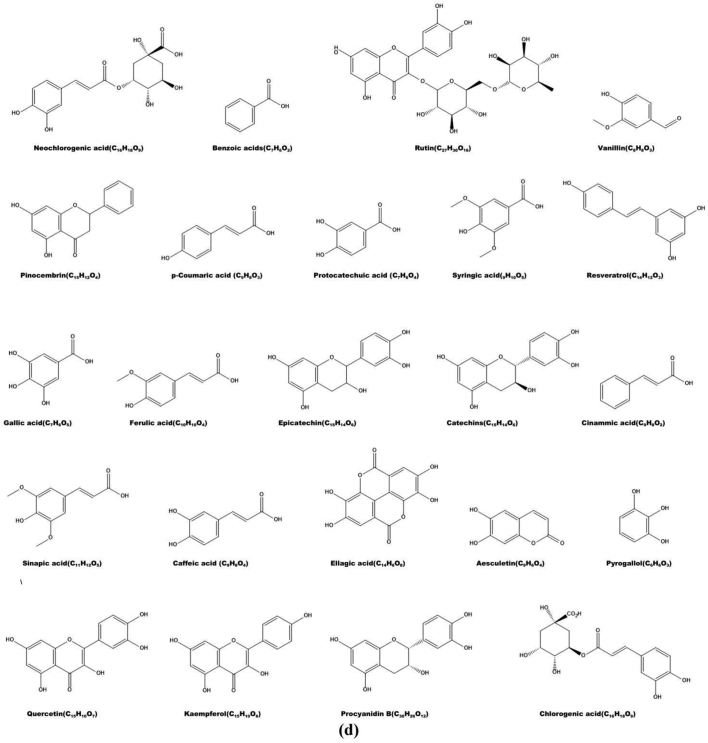
The chemical and structural formulas of nutritionally important chemicals found in *M. germanica* fruit. All formulas are drawn with ChemDraw Ultra 12.0 (Cousins, 2011). a) Organic acids. b) Sugars. c) The fatty acids. d) Phenolic compounds.

According to published literature, the most common organic acids found in *M. germanica* fruits are succinic, oxalic, citric, quinic, malic, fumaric, and tartaric acids (Fig. 3a) (Cosmulescu et al., 2020; Cosmulescu & Scrieciu, 2020; Glew, Ayaz, Sanz, et al., 2003a; Glew, Ayaz, Sanz, et al., 2003b; Glew, Ayaz, Vanderjagt, et al., 2003; Ozturk et al., 2019; Selcuk & Erkan, 2015a; Selcuk & Erkan, 2015b; Tessa et al., 2021;Voaides et al., 2021; Voronkov, Ivanova, et al., 2020). The quantity of specific acids present in the fruit depends on its maturity and the duration of storage after harvesting, in addition to the climate and production methods. Overripe fruits, after a prolonged period of storage, tend to have lower concentrations of organic acids (Famiani et al., 2015; Glew, Ayaz, Sanz, et al., 2003a; Selcuk & Erkan, 2015a; Selcuk & Erkan, 2015b), which can be minimized by storing them in a palliflex-controlled environment (Donno et al., 2017). This helps maintain the organic acid levels, which in turn improves the fruit's overall flavour and nutritional value (Popovic-Djordjevic et al., 2023; Selcuk & Erkan, 2015a; Selcuk & Erkan, 2015b; Voaides et al., 2021). The type and quantity of organic acids present in the fruit also have a significant impact on its organoleptic properties, including its flavour, shape, and aroma (Batista-Silva et al., 2018; Bibalani & Mosazadeh-Sayadmahaleh, 2012;Popovic-Djordjevic et al., 2023; Voaides et al., 2021).

#### 4.1.2 Sugars

Medlar fruit is a rich source of carbohydrates (Al-Amoudi et al., 2019; Cevahir & Bostan, 2021; Famiani et al., 2015; Pourmortazavi et al., 2005; Selcuk & Erkan, 2015a; Żołnierczyk et al., 2021). Fresh fruits contain anywhere between 23 and 73% carbs, with the primary sugars being sucrose, fructose, and glucose (Cevahir & Bostan, 2021; Donno et al., 2017; Glew, Ayaz, Sanz, et al., 2003b). The primary sugars contained in *M. germanica* fruit are shown in Table 5: sucrose, fructose, and glucose (Fig. 3b) (Al-Amoudi et al., 2019; Aydin & Kadioglu, 2001; Donno et al., 2017; Glew, Ayaz, Sanz, et al., 2003b; Popovic-Djordjevic et al., 2023; Selcuk & Erkan, 2015a; Voaides et al., 2021). Aydin and Kadioglu (2001) conducted a study on the levels of soluble sugars in medlar fruit and found that sorbitol, a sugar alcohol, is commonly used in sugar-free meals and diabetic products due to its pleasant taste, low glycemic index, and anti-hyperglycemic properties (Al-Amoudi et al., 2019; Msomi et al., 2021; Pető et al., 2016; Popovic-Djordjevic et al., 2023; Sebek et al., 2019; Voaides et al., 2021; Żołnierczyk et al., 2021).

Pectins extracted from medlar fruit contain L-arabinose, D-galacturonic acid, d-glucose, L-rhamnose, and D-galactose (Al-Amoudi et al., 2019; Popovic-Djordjevic et al., 2023; Stankovic et al., 2022). Sugar concentration, like organic acids concentration, is influenced by various factors such as cultivars, the genotype, production region, growth and maturation phases (Aydin & Kadioglu, 2001; Popovic-Djordjevic et al., 2023; Stankovic et al., 2022; Voaides et al., 2021). The ripening stage of *M. germanica* fruit is characterized by an increase in fructose and glucose content, and a decrease in sucrose content, while organic acid concentration declines. and a decrease in sucrose content, while organic acid concentration declines (Aydin & Kadioglu, 2001; Batista-Silva et al., 2018; Glew, Ayaz, Sanz, et al., 2003b). After harvesting, the chemical structure of the major sugars in the fruit changes progressively (Cevahir & Bostan, 2021; Glew, Ayaz, Vanderjagt, et al., 2003; Popovic-Djordjevic et al., 2023).

#### 4.1.3 Volatile compounds

Fruit taste is contributed to by volatile scent molecules, even though they exist in very small amounts (Ercisli, 2007;Pourmortazavi et al., 2005; Veličković et al., 2013). Fragrance is predominantly associated with lipid molecules during fruit ripening, which are generally assumed to act as progenitors for volatile compounds (Pourmortazavi et al., 2005; Veličković et al., 2013). A study on medlar fruits found that C6 alcohols ((Z)-3-hexenol, hexanol) and aldehydes (furfural, (*E*)-2-hexenal, hexanal) and alcohols were the primary volatile components in the fruits (Popovic-Djordjevic et al., 2023; Sadeghinejad et al., 2022; Stankovic et al., 2022; Yildiz et al., 2023). The study also discovered that (E)-2-hexenal and hexanal reduced in mature fruit, while furfural level increased significantly in ripe fruits. However, the amount of alcohol in ripe fruits increased (Davoodi et al., 2018; Popovic-Djordjevic et al., 2023; Pourmortazavi et al., 2005; Veličković et al., 2013).

Fatty acids as well as their ester forms were produced throughout the ripening process (Veličković et al., 2013). P-cymene, γ-terpinene, terpinene-4-ol, and γ-eudesmol were the predominant terpenes found in both unripe and mature fruits (Popovic-Djordjevic et al., 2023; Pourmortazavi et al., 2005). Phellandrene and γ-terpinene were found in significant levels solely in fruits, whereas additional terpenes were found in negligible volumes (Davoodi et al., 2018; Popovic-Djordjevic et al., 2023; Veličković et al., 2013). Tessa et al. (2021) found that the most frequent volatile compounds in mature fruit were phellandrene and γ-terpinene. Only three volatile chemicals were found in medlar seeds: benzaldehyde, tetradecane, and pentadecane (Pourmortazavi et al., 2005; Veličković et al., 2013; Popovic´-Djordjevic et al., 2023; Voaides et al., 2021). Medlar fruits possess tetraterpenoids that include carotenoids along with volatile terpenes (Gülçin et al., 2011). The most prominent of them is β-carotene (Popovic-Djordjevic et al., 2023; Voaides et al., 2021). Carotenoids contain antioxidant properties and have been linked to a reduced probability of various ailments such as cancer, macular degeneration, and brain damage (Olives Barba et al., 2006; Popovic-Djordjevic et al., 2023; Voaides et al., 2021).

#### 4.1.4 Fatty acids

The classification of fatty acids as either unsaturated or saturated is determined by the presence or lack of double bonds (Azizi & Azizi, 2011; Voronkov, Ivanova, et al., 2020; (Glew, Ayaz, Sanz, et al., 2003b). Naturally occurring unsaturated fatty acids have cis double-bonded configurations, while trans configurations in products are the result of technological processing (Popovic-Djordjevic et al., 2023; Voronkov, Ivanova, et al., 2020). Omega-6 and Omega-3 polyunsaturated fatty acids are essential nutrients that must be obtained through dietary intake (Voaides et al., 2021; Voronkov, Ivanova, et al., 2020). These two fatty acids have been classified as nutritional foods and nutraceuticals because of their crucial role in metabolic processes that result in health benefits (Dąbrowska et al., 2019; Gülçin et al., 2011; Murathan et al., 2016; Orsavova et al., 2015; Popovic-Djordjevic et al., 2023; Voronkov, Ivanova, et al., 2020). Mature fruits contain saturated fatty acids such as hexanoic (caproic), dodecanoic (lauric), tetradecanoic (myristic), and pentadecanoic (pentadecylic). Palmitic acid is present in both unripe and ripe fruits (Glew, Ayaz, Sanz, et al., 2003b; Orsavova et al., 2015; Veličković et al., 2013; Voronkov, Ivanova, et al., 2020).

In the literature, stearic, oleic, linoleic, arachidic, behenic, and lignoceric acids have also been described (Popovic-Djordjevic et al., 2023; Voronkov, Ivanova, et al., 2020). The key saturated and unsaturated fatty acids (linolenic, linoleic, oleic, palmitic, and stearic acids) were discovered by Glew, Ayaz, Sanz, et al. (2003b). In addition, the esters ethyl-oleate and ethyl-hexadecanoate were detected in mature medlar fruit, demonstrating the biogenetic synthesis of saturated and unsaturated fatty acids during the stage of ripening, as well as their transformation to the appropriate esters (Fig. 3c) (Veličković et al., 2013).

#### 4.1.5 Vitamins and other biogenic elements

Fruits are a rich source of vitamins C, E, A, and B9 (folacin), B6 (pyridoxine), B3 (niacin), and B1 (thiamine) (Donno et al., 2017; Voaides et al., 2021). These vitamins, unlike macronutrients such as proteins, carbohydrates, and lipids, are not required for energy production (Ercisli et al., 2012; Popovic-Djordjevic et al., 2023). However, vitamins play a crucial role in cellular consumption as co-enzymes and are involved in the release and storage of energy (Rop et al., 2011). Vitamins A, C, and E are well known for their antioxidant activities (Cevahir & Bostan, 2021; Ercisli et al., 2012; Kader, 2000; Stankovic et al., 2022). Among medlar fruits, vitamin C (Ascorbic acid), vitamin E (α-tocopherol), and dehydroascorbic acid are the most investigated vitamins, according to Table 5 (Akbulut et al., 2016; Cevahir & Bostan, 2021; Cosmulescu et al., 2020; Cosmulescu & Scrieciu, 2020; Ercisli et al., 2012; Glew, Ayaz, Sanz, et al., 2003b; Glew, Ayaz, Vanderjagt, et al., 2003; Gülçin et al., 2011; Popovic-Djordjevic et al., 2023).

In human nutrition, major elements such as P, Ca, Mg, and K, and trace elements such as Zn, Fe, Cu, and Mn are essential for normal biological system activity. Although these elements cannot be ingested in large amounts, they need to be included regularly in the diet (Glew, Ayaz, Vanderjagt, et al., 2003; Pető et al., 2016; Popovic-Djordjevic et al., 2023; Stankovic et al., 2022). Major elements such as P and Ca are present in bones and teeth, while Mg acts as a relaxant, K controls the balance of fluids and electrolytes and supports the nervous system function (Ercisli et al., 2012; Pető et al., 2016). Trace elements, on the other hand, are required for several enzymes, and they play an essential role in various biological functions, including adequate development and growth during childbirth, growing up, and adulthood (Abbaspour et al., 2014; Cashman, 2002; Pető et al., 2016; Pohl et al., 2013; Popovic-Djordjevic et al., 2023; Rop et al., 2011).

Medlar fruit is rich biogenic components such as Mg, K, Na, P, Fe, and Ca (Pető et al., 2016). The fruit also contains other physiologically essential elements such as Mn, Co, S, Zn, Mo, and Cu (Ercisli et al., 2012; Glew, Ayaz, Vanderjagt, et al., 2003; Pető et al., 2016). Many investigations found K to be the utmost prevalent component, followed by Mg, Na, and Ca (Azizi & Azizi, 2011; Ercisli et al., 2012; Glew, Ayaz, Vanderjagt, et al., 2003; Gülçin et al., 2011; Kalyoncu et al., 2013; Pető et al., 2016; Popovic-Djordjevic et al., 2023; Rop et al., 2011; Żołnierczyk et al., 2021). The chemical composition of medlar fruit is regulated by both variety and maturation stages (Ercisli et al., 2012; Glew, Ayaz, Vanderjagt, et al., 2003; Pető et al., 2016; Yildiz et al., 2023). Macroelements such as Mg, K, and Ca exhibit a significant drop in concentration throughout fruit maturity (Ercisli et al., 2012; Glew, Ayaz, Vanderjagt, et al., 2003; Popovic-Djordjevic et al., 2023; Rop et al., 2011). Medlar fruit has more biogenic elements (Mg, K, Fe, P, and Ca) than conventional fruits like apples, bananas, grapes, and mangoes (Ercisli et al., 2012; Popovic-Djordjevic et al., 2023; Rop et al., 2011).

#### 4.1.6 *M. germanica* phenolic profile

Minerals and vitamins are essential components found in fruits that provide nutritional benefits, while phenolic compounds and carotenoids are non-nutritive components known for their medicinal properties that improve human health (Kader, 2000; Sadeghinejad et al., 2022; Shahidi, Janitha, & Wanasundara, 1992). Medlar fruit is a great source of polyphenols, including flavonoids, coumarins, and phenolics (Gruz et al., 2011; Gülçin et al., 2011; Haminiuk et al., 2012; Islam et al., 2019; Sadeghinejad et al., 2022; Shahidi et al., 1992; Usman et al., 2022), as shown in Fig. 3d (Gruz et al., 2011; Gülçin et al., 2011; Rop et al., 2011; Sadeghinejad et al., 2022). Despite limited literature, Table 5 shows that medlar fruit contains a substantial amount of these beneficial compounds (Akbulut et al., 2016; Gruz et al., 2011; Gülçin et al., 2011; Ozturk et al., 2019; Popovic-Djordjevic et al., 2023; Rop et al., 2011; Sadeghinejad et al., 2022; Stankovic et al., 2022; Tessa et al., 2021; Voaides et al., 2021; Żołnierczyk et al., 2021).

Recent studies have also revealed that medlar fruits contain aesculetin and pinocembrin, which are isoflavonoid and flavonoid compounds, respectively (Akbulut et al., 2016; Gruz et al., 2011; Gülçin et al., 2011; Stankovic et al., 2022). Polyphenols are known for their antioxidant properties and have been proven to help prevent heart disease, fractures, neurological diseases, cancers, and diabetes (Donno et al., 2017; Popovic-Djordjevic et al., 2023; Żołnierczyk et al., 2023). Some flavonoids, including epicatechin gallate, rutin, quercetin, quercetin-3-O-a-l-rhamnopyranoside, luteolin, and other compounds, as well as some phenolic acids, have been found to inhibit the activity of α-glucosidase, a carbohydrate-digesting enzyme (Al-Ishaq et al., 2019; Assefa et al., 2020; Isbilir et al., 2019; Pourmortazavi et al., 2005; Voaides et al., 2021). The main components of medlar fruits are benzoic acids (such as gallic acid, protocatechuic acid, p-aminobenzoic acid, salicylic acid, (3,4)-hydroxybenzoic acid, syringic acid, etc.) and cinnamic acids (such as caffeic acid, p-coumaric acid, neochlorogenic acid, chlorogenic acid, ferulic acid, etc.) as listed in Table 5.

Among the discovered phenolic acids chlorogenic acid was the most prevalent in numerous investigations (Akbulut et al., 2016; Gülçin et al., 2011; Ozturk et al., 2019; Popovic-Djordjevic et al., 2023; Sadeghinejad et al., 2022; Stankovic et al., 2022; Tessa et al., 2021; Voaides et al., 2021; Żołnierczyk et al., 2021). The quantities of phenolic acids were shown to decline throughout the process of maturation (Akbulut et al., 2016; Gruz et al., 2011; Gülçin et al., 2011).

## 5 Patents

**Fig. 4:**
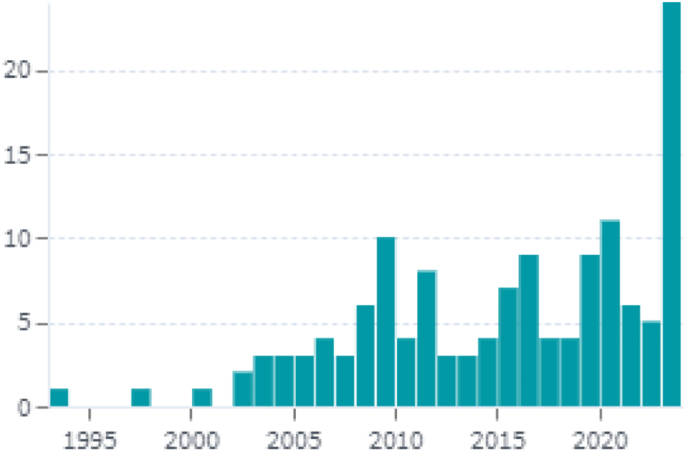
Shows patent applications per year.

**Fig. 5:**
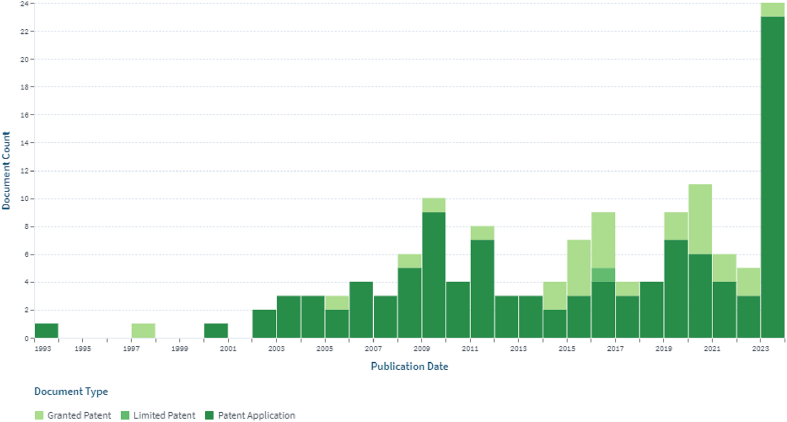
Patent applications status.

**Fig. 6:**
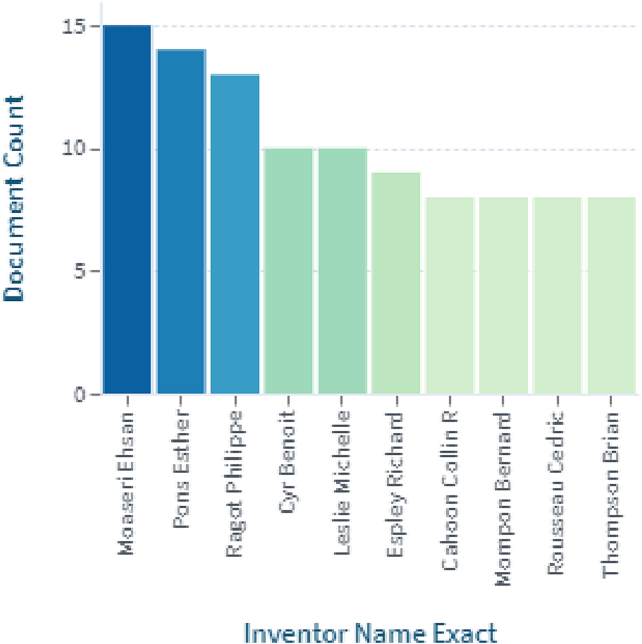
Inventors of Patent applications.

A search by “*Mespilus germanica*” indicated 138 patents belonging to 69 simple and 57 extended families. Patents over time, patent application status and inventor's names are shown in Fig. 4, Fig. 5, Fig. 6 respectively (https://www.lens.org/lens/search/patent/list?q=Mespilus%20germanica). A patent granted to Todor and Irena (2016) indicates use of its fruits along with peach, *Mespilus germanica*, rosehip, persimmon, orange, clementine, grapefruit, kiwi, and banana fruits for use as a facial mask, having beneficial skin properties, especially for acneic skin or for other problems. The product has as deep soothing and cleansing effect, it absorbs excess oils and moisture from the skin, revitalizes and detoxes and has active anti-irritation and antibacterial properties. Similarly an invention is based on a gluten-free mill type chestnut-base product where each component including medlar has a protective and supportive effect on the health of people of all ages, with high nutritional value, produced in accordance with the technique of production of national and international supplementary foods (Elif et al., 2023).

## 6 Conclusion

This review discusses the increasing popularity of *M. germanica* L. due to its unique fruit characteristics and potential health benefits. While traditional medicine has employed various parts of the plant for medicinal purposes, there is a lack of extensive scientific investigation into its chemical components and physiological traits. The plant's flavonoids require further investigation, and there have been limited inquiries into the use of medlar waste. The study highlights the fruit's potential application in the food industry for the creation of valuable meals and novel goods. Acquiring knowledge about the nourishing, biological, and therapeutic properties of *M. germanica* fruits is crucial for rekindling interest in this exceptional fruit tree and reviving its cultivation and use.

## CRediT authorship contribution statement

**Doru Ion Nistor:** Writing – original draft. **Romina Alina Marc:** Writing – review & editing, Visualization, Project administration, Conceptualization. **Crina Carmen Mureșan:** Visualization, Validation, Supervision.

## Declaration of competing interest

The authors declare that there is no conflict of interest.

## Data Availability

No data was used for the research described in the article.
